# Understanding adherence in virally suppressed and unsuppressed human immunodeficiency virus-positive urban patients on second-line antiretroviral treatment

**DOI:** 10.4102/sajhivmed.v21i1.1107

**Published:** 2020-08-11

**Authors:** Siphamandla B. Gumede, Willem D.F. Venter, Samanta T. Lalla-Edward

**Affiliations:** 1 Ezintsha, a sub-division of Wits Reproductive Health and HIV Institute, University of the Witwatersrand, Johannesburg, South Africa; 2 Department of Interdisciplinary Social Science, Public Health, Utrecht University, Utrecht, The Netherlands

**Keywords:** adherence, viral load suppression, virological failure, antiretroviral therapy, South Africa

## Abstract

**Background:**

Understanding antiretroviral therapy (ART) adherence may assist in designing effective support interventions.

**Objectives:**

This study elicited perspectives on how to promote treatment adherence from virologically suppressed and unsuppressed patients receiving second-line ART.

**Methods:**

This was a cross-sectional study conducted with randomly selected patients active on second-line ART, from five public health facilities in the Johannesburg inner city. Data were collected on demographics, clinical information, participant’s experiences and ART knowledge. Virological failure was defined as exceeding 1000 copies/mL.

**Results:**

The study sample comprised 149 participants; of which 47.7% (*n* = 71) were virally unsuppressed and 69.1% (*n* = 103) were women; the median age of the participants was 42 years (interquartile range [IQR] 36–47 years). Experiencing medication-related difficulties in taking second-line ART (*p* = 0.003), finding second-line regimen more difficult to take than a first-line regimen (*p* = 0.001) and experiencing side effects (*p* < 0.001) were all subjective predictors of virological failure. Participants’ recommendations for improving adherence included the introduction of a single tablet regimen (31.6%, *n* = 55), reducing the dosage to once daily (26.4%, *n* = 46) and reducing the pill size for second-line regimen (4.0%, *n* = 7).

**Conclusion:**

The results of this study highlight the importance of improving patients’ knowledge about adherence and motivation to continue ART use despite the persistence of side effects and difficulties with taking medication.

## Introduction

The World Health Organization (WHO) defines adherence as the degree to which a patient is able to follow a treatment plan and take medication at prescribed times.^1^

Factors that affect treatment adherence include changes in daily routines, forgetting to take medication, side effects, depression, being away from home, comorbidity, lack of knowledge and desire to take treatment.^2,3,4^ In addition, patients experiencing financial constraints, social issues such as the fear of disclosure and lack of understanding of treatment benefits are more prone to non-adherence to treatment and illnesses.^5,6^ Some studies have reported disclosure and relationship with the person being disclosed to as predictors of adherence.^7,8^

The South African government’s adherence promotion strategies include routine viral load monitoring, adherence counselling, pill counting, adherence clubs and routine completion of clinical stationery.^9,10,11^ Despite all these antiretroviral therapy (ART) adherence strategies being implemented, treatment failure amongst patients on first- and second-line ART remains an issue.^12^ Lapses in ART medication adherence can lead to viral rebound with ongoing immunosuppression and viral resistance.^1,13,14^ However, not much is understood about the perspectives of patients regarding adherence challenges.^15,16^

Therefore, this study sought to describe and obtain perspectives on treatment adherence from two separate groups of patients on second-line therapy, those who were suppressed and those who were not, to understand treatment-taking behaviour.

## Materials and methods

### Study design and setting

This was a cross-sectional study conducted between July and August 2018 in a sub-population of patients receiving second-line ART at the end of June 2018. Five public health facilities in inner-city Johannesburg (two hospitals, one community health centre and two primary healthcare clinics) were included in the study.

### Study population

The study population comprised patients aged 18 years and older who were on second-line ART for at least 1 month or longer.

### Data collection

#### Sample selection and recruitment

We randomly sampled 10% of the population of 1500 eligible patients. The total number of active patients on second-line treatment per facility was divided by the total sample size (*n* = 150) to determine the interval that needed to be used to select the eligible patients. Using this formula, every *n*th (different for each facility) record from the register or list of active patients on second-line treatment in each facility was selected and recruited to the study until the facility sample size was reached. Once the eligible patients were identified, they were invited to participate in the study in one of the two ways: telephonically or in facility recruitment where researchers met them at the facility during their scheduled clinic visit. For the patients who refused to participate in the study, the next *n*th patient was recruited.

#### Data collection, tool and variables

A pretested semi-structured questionnaire was used, which consisted of five sections: (1) demographic data, (2) comorbidity information, (3) human immunodeficiency virus (HIV) diagnosis and care information, (4) experiences on the first-line regimen and adherence and (5) experiences on second-line regimen and adherence. Information collected included demographic information (facility name, sex, age, relationship status, employment status and education level), comorbidity information, experiences on both first- and second-line treatment, disclosure information, duration on ART, reasons for starting ART, side effects, self-reported treatment interruptions, challenges with taking second-line treatment, treatment supporter information and insights into how adherence could be improved.

#### Questionnaire administration

Data were collected by the principal investigator and a trained research assistant. The interviews were conducted in English as it was the most commonly spoken language in the study setting and all participants could speak it.

#### Data entry, cleaning and analysis

Data were captured into REDCap immediately after interviews were conducted. The research team conducted data clean-up by running data quality checks in REDCap and STATA (quantitative data). For the closed-ended questions, we assessed the association between outcome variables and selected socio-demographic and health-related characteristics. Pearson’s chi-squared test was used to assess trend associations between categorical variables. Continuous data were summarised and analysed using the median and interquartile ranges (IQRs). Logistic and multiple logistic regression models (bivariate and multivariate logistic regression) were built for key outcome variables, such as viral load, difficulties in taking second-line regimen and side effects, to identify independent predictors. We reported unadjusted and adjusted odds ratios (ORs), 95% confidence interval (CI) and *p*-values – *p*-values that were less than 0.05 were considered significant. Open-ended questions were analysed using qualitative data analysis methods. Data were coded and thematic analysis was performed. Where appropriate, quotations have been included to support the reported results.

### Ethical consideration

Ethical approval to conduct the study was obtained from the University of the Witwatersrand Human Research Ethics Committee (ethical clearance number: M170691). Approval was also granted by the Johannesburg Health District (DRC Ref No. 2017-08-003 and NHRD Ref No. GP_201708_030). Participants were informed that participation in the study was voluntary and that refusal would not affect their relationship with their healthcare provider or facility. All patients who agreed to participate in the study signed an informed consent form. To ensure confidentiality, there were no linkages between the data collected in the questionnaire and the patients’ clinic information. Participants were reimbursed for their travel.

## Results

### Sample characteristics

A total of 150 out of 1500 active patients on second-line ART across the five public health facilities were interviewed (69.1%, *n* = 103 women). During the quality checking processes, we found that one of the participants was younger than 18 years and was subsequently omitted from the analysis. The median age of the participants was 42 years (IQR 36–47 years). Most of the participants were single (38.1%, *n* = 57); 30.2% (*n* = 45) participants were married. Nearly two-thirds of the participants were born in South Africa (61.1%, *n* = 91), whilst almost one-third of the participants were born in Zimbabwe (32.9%, *n* = 49). The majority (87.2%, *n* = 130) of participants had completed at least their secondary or high school-level education. A minority (8.1%, *n* = 12) of participants had completed tertiary qualifications; 4.7% (*n* = 7) participants had never attended a school. Of the total participants, 45.6% (*n* = 68) were unemployed. The majority of participants were identified as Christian (87.9%, *n* = 131). Hypertension (65.1%, *n* = 28/43), diabetes (9.3%, *n* = 4/43) and hypercholesterolaemia (9.3%, *n* = 4/43) were the most common concomitant conditions reported by the participants. The average distance travelled to reach a health facility was 5 km (IQR: 2 km – 15 km), with 57.7% (*n* = 86) participants travelling 5 km or less to reach the health facility.

### Socio-demographic and virological status

Table 1 shows the socio-demographic characteristics disaggregated by virological status. Nearly half (47.7%, *n* = 71) of the participants interviewed had virological failure (VLF), with women accounting for 73.2% (*n* = 52). With regard to age, of the total unsuppressed participants, 39.4% (*n* = 28) were between 30 and 39 years. Of the participants with virological suppression (VLS), 29.5% (*n* = 23) had comorbidity compared to VLF participants (28.2%, *n* = 20).

**TABLE 1:** Socio-demographic and treatment-taking characteristics disaggregated by virological status of second-line participants in five health facilities in the Johannesburg inner city.

Variable Total patient or participants recruited	Total *N* = 149 (100)		Virological status				*p*
			Suppressed (VLS), 78 (52.3)		Unsuppressed (VLF), 71 (47.7)		
	*n*	%	*n*	%	*n*	%	
**Facility**							0.001*
Charlotte Maxeke Johannesburg Academic Hospital	32	21.5	19	24.4	13	18.3	
Hillbrow Community Health Centre	86	57.7	43	55.1	43	60.6	
South Rand Hospital	21	14.1	16	20.5	5	7.0	
Primary Healthcare Clinics (Malvern Clinic Yeoville Clinic†)	10	6.7	0	0.0	10	14.1	
**Sex**							0.300
Female	103	69.1	51	65.4	52	73.2	
Male	46	30.9	27	34.6	19	26.8	
**Age, Median (42, IQR: 35–47) (years)**							0.152
< 30	11	7.4	3	3.9	8	11.3	
30–39	52	34.9	24	30.8	28	39.4	
40–45	43	28.9	25	32.1	18	25.4	
45+	43	28.9	26	33.3	17	23.9	
**Country of birth**							0.085
South Africa	91	61.1	42	53.9	49	69.0	
Zimbabwe	49	32.9	32	41.0	17	23.9	
Other	9	6.0	4	5.1	5	7.0	
**Home language**							0.893
Zulu	62	41.6	32	41.0	30	42.3	
Ndebele	36	24.2	21	26.9	15	21.1	
Xhosa	12	8.1	6	7.7	6	8.5	
Sotho	12	8.1	5	6.4	7	9.9	
Other	27	18.1	14	18.0	13	18.3	
**Relationship status**							0.310
Married	45	30.2	28	35.9	17	23.9	
Cohabiting	38	25.5	16	20.5	22	31.0	
Single	57	38.3	30	38.5	27	38.0	
Other	9	6.0	4	5.1	5	7.0	
**Highest education level completed**							0.737
Never went to school	7	4.7	4	5.1	3	4.2	
Secondary or high school	130	87.2	69	88.5	61	85.9	
Tertiary	12	8.1	5	6.4	7	9.9	
**Employment status**							0.613
Employed	65	43.6	37	47.4	28	39.4	
Unemployed	68	45.6	33	42.3	35	49.3	
Other‡	16	10.7	8	10.3	8	11.3	
**Religion**							0.098
Christian	131	87.9	67	85.9	64	90.1	
Ancestral/traditional	8	5.4	7	9.0	1	1.4	
Other	10	6.7	4	5.1	6	8.5	
**Comorbidity**							0.859
Yes	43	28.9	23	29.5	20	28.2	
No	106	71.1	55	70.5	51	71.8	
**Distance travelled to the facility,**							0.764
Median (5, IQR: 2–15)							
**5 km or less**	86	57.7	45	57.7	41	57.8	
6 km – 10 km	21	14.1	12	15.4	9	12.7	
11 km – 20 km	23	15.4	10	12.8	13	18.3	
Above 20 km	19	12.8	11	14.1	8	11.3	
**Who was the first person to whom you disclosed your HIV status?**							0.228
Partner	70	47.0	33	42.3	37	52.1	
Family or relative member	77	51.7	43	55.1	34	47.9	
Friend	2	1.3	2	2.6	0	0.0	
**How long did it take you to disclose your HIV status?**							0.306
1 week	94	63.1	48	61.5	46	64.8	
1–2 weeks	8	5.4	4	5.1	4	5.6	
3–4 weeks	4	2.7	4	5.1	0	0.0	
More than 4 weeks	42	28.2	22	28.2	20	28.2	
Never disclosed	1	0.7	0	0.0	1	1.4	
**Do you have anyone supporting you in taking your ARVs currently?**							0.755
Yes	118	79.2	61	78.2	57	80.3	
No	31	20.8	17	21.8	14	19.7	
**Do you feel the current regimen is difficult to take compared to the previous regimen(s)?**							0.001*
Yes	78	52.3	31	39.7	47	66.2	
No	71	47.7	47	60.3	24	33.8	
**Have you been experiencing difficulties in taking the current regimen?**							0.003*
Yes	57	38.3	21	26.9	36	50.7	
No	92	61.7	57	73.1	35	49.3	
**Have you experienced any side effects since you switched regimens or drugs?**							< 0.001*
Yes	71	47.7	26	33.3	45	63.4	
No	78	52.3	52	66.7	26	36.6	
**Have you ever stopped taking the current regimen for over a month?**							0.728
Yes	5	3.4	3	3.8	2	2.8	
No	144	96.6	75	96.2	69	97.2	
**Have you ever felt like stopping the current regimen or drugs completely?**							0.642
Yes	15	10.1	7	9.0	8	11.3	
No	134	89.9	71	91.0	63	88.7	
**Have you ever felt like switching current regimen or drugs for something else?**							0.142
Yes	58	38.9	26	33.3	32	45.1	
No	91	61.1	52	66.7	39	54.9	

*N*, number; VLS, virological suppression; n, number; VLF, virological failure; *p*, probability value; IQR, interquartile range; km, kilometre; ARVs, antiretrovirals; HIV, human immunodeficiency virus.

† , Second-line patients are often managed at a higher level of facility.

‡ , People not looking for employment at that time, for example, students and housewives.

* , *p* < 0.05.

### Disclosure, treatment support and virological status

Almost all the participants’ first disclosure of their HIV status was to a partner or relative (98.7%, *n* = 147 combined). More VLS participants (55.1%, *n* = 43) disclosed about their HIV status to a family member first, whilst most VLF participants (52.1%, *n* = 37) chose to disclose to their partners first. Almost no disclosure to friends was reported. Disclosure did not show any statistical significance. Participants typically (63.1%, *n* = 94) disclosed within 1 week after HIV diagnoses, with more VLF participants reporting early disclosure than VLS participants (64.8%, *n* = 46 vs. 61.5%, *n* = 48). Most participants (79.2%, *n* = 118) had treatment supporters (VLF = 80.3%, *n* = 57; VLS = 78.2%, *n* = 61). Whilst 10.1% (*n* = 15) of participants felt like stopping treatment completely at some point, only 3.4% (*n* = 5) stopped treatment for longer than 1 month, more in VLS participants, although this was not statistically significant.

### Factors of virological failure and adherence

Overall, there were more participants (52.3%, *n* = 78) who felt that taking a second-line regimen was difficult compared to the first-line regimen, with the VLF group (66.2%, *n* = 47, *p* = 0.001) predominantly reporting this challenge. Generally, 38.3% (*n* = 57/149) experienced difficulties in taking the second-line regimen (*p* = 0.003). Of these, about two-thirds (63.2%, *n* = 36) were VLF participants (*p* = 0.003). Just under half (47.7%, *n* = 71/149) of the participants experienced side effects whilst taking their second-line regimen, and of these, 63.4% (*n* = 45) were VLF participants (*p* < 0.001).

Table 2 presents results from both bivariate and multivariate logistic regression analysis. No association was detected between VLF and relationship status in bivariate analysis. However, in multivariate analysis, participants who cohabit were three times more likely to have a VLF than those who are married (adjusted odds ratios [AORs] 3.1, 95% CI = 1.1–8.9; *p* = 0.035). Unemployed participants were two and a half times more likely to have treatment-related side effects compared to employed participants (AOR 2.5, 95% CI = 1.1–5.7; *p* = 0.023). Results for age did not show any statistical significance but older people were less likely to be unsuppressed.

**TABLE 2:** Logistic regression analysis between outcome variables and socio-demographic characteristics of second-line participants in Johannesburg inner city.

Variable	Virological failure				Experiencing difficulties taking second-line regimen				Feeling that second-line regimen is difficult to take				Side effects			
	UOR	*p*	AOR	*p*	UOR	*p*	AOR	*p*	UOR	*p*	AOR	*p*	UOR	*p*	AOR	*p*
**Facility**																
CMJAH	Ref	-	Ref	-	Ref	-	Ref	-	Ref	-	Ref	-	Ref	-	Ref	-
HCHC	1.3 (0.6–3.1)	0.460	1.6 (0.6–4.4)	0.475	2.0 (0.8–4.7)	0.113	2.0 (0.7–5.7)	0.185	2.4 (1.1–5.6)	0.037*	1.7 (0.7–4.8)	0.246	2.1 (0.9–4.8)	0.080	1.8 (0.7–4.9)	0.233
SRH	0.4 (0.1–1.4)	0.168	0.4 (0.1–1.4)	0.136	0.3 (0.1–1.5)	0.170	0.4 (0.1–2.1)	0.301	0.7 (0.2–2.2)	0.503	0.6 (0.2–2.4)	0.505	0.7 (0.2–2.2)	0.503	0.5 (0.1–1.8)	0.266
PHC (Yeoville and Malvern)	1	-	1	-	0.9 (0.2–4.4)	0.941	0.7 (0.1–3.8)	0.631	15.0 (1.7–133.6)	0.015*	8.6 (0.8–87.7)	0.069	1.7 (0.4–7.0)	0.484	1.7 (0.3–8.8)	0.538
**Age (years)**																
< 30	Ref	-	Ref	-	Ref	-	Ref	-	Ref	-	Ref	-	Ref	-	Ref	-
30–39	0.4 (0.1–1.8)	0.259	0.6 (0.1–2.9)	0.496	0.7 (0.2–2.6)	0.614	0.8 (.2–3.1)	0.717	1.2 (0.3–4.6)	0.815	1.6 (0.3–7.6)	0.551	1.0 (0.3–3.6)	0.966	1.2 (0.3–5.0)	0.786
40–45	0.3 (0.06–1.2)	0.079	0.5 (0.1–2.9)	0.462	0.5 (0.1–1.9)	0.301	0.8 (.2–3.3)	0.717	0.5 (0.1–1.8)	0.256	0.6 (0.1–3.2)	0.597	0.8 (0.2–3.0)	0.736	1.7 (0.4–7.4)	0.486
45+	0.2 (0.06–1.1)	0.059	0.6 (0.1–3.4)	0.595	0.3 (0.1–1.1)	0.074	0.5 (0.1–2.1)	0.327	0.4 (0.1–1.5)	0.160	0.7 (0.1–3.3)	0.612	0.5 (0.1–1.9)	0.301	0.9 (0.2–4.3)	0.935
**Gender**																
Female	Ref	-	Ref	-	Ref	-	Ref	-	Ref	-	Ref	-	Ref	-	Ref	-
Male	0.7 (0.3–1.4)	0.301	0.9 (0.4–2.1)	0.776	0.6 (0.3–1.3)	0.191	0.5 (0.2–1.2)	0.139	0.5 (0.3–1.1)	0.073	0.6 (0.3–1.3)	0.244	0.7 (0.3–1.4)	0.301	0.9 (0.4–2.1)	0.857
**Relationship status**																
Married	Ref	-	Ref	-	Ref	-	Ref	-	Ref	-	Ref	-	Ref	-	Ref	-
Single	1.5 (0.7–3.3)	0.332	2.1 (0.8–5.4)	0.136	1.5 (0.7–3.4)	0.336	1.3 (0.5–3.2)	0.598	1.1 (0.5–2.5)	0.777	0.9 (0.4–2.2)	0.799	2.0 (0.9–4.5)	0.087	2.0 (0.8–4.8)	0.143
Cohabiting	2.3 (0.9–5.5)	0.069	3.1 (1.1–8.9)	0.035*	1.6 (0.7–4.0)	0.300	1.2 (.4–3.2)	0.751	1.0 (0.4–2.3)	0.949	0.7 (0.2–1.9)	0.470	2.0 (0.8–4.9)	0.120	2.2 (0.8–5.9)	0.134
Other	2.1 (0.5–8.7)	0.328	2.4 (0.5–13.1)	0.301	1.8 (0.4–7.6)	0.442	2.9 (0.5–16.3)	0.238	0.3 (0.05–1.3)	0.105	0.3 (0.04–1.8)	0.170	2.3 (0.5–9.7)	0.269	2.7 (0.5–14.2)	0.239
**Highest education level completed**																
Secondary/High school	Ref	-	Ref	-	Ref	-	Ref	-	Ref	-	Ref	-	Ref	-	Ref	-
Tertiary	1.6 (0.5–5.3)	0.452	2.2 (0.5–9.3)	0.279	1.1 (0.3–3.8)	0.827	0.9 (0.2–3.6)	0.897	0.6 (0.2–2.0	0.422	0.8 (0.2–3.3)	0.764	1.1 (0.3–3.7)	0.838	1.1 (0.3–4.1)	0.943
Never went to school	0.9 (0.2–3.9)	0.834	0.4 (0.1–3.3)	0.421	0.6 (0.1–3.4)	0.602	0.8 (0.1–5.3)	0.854	0.6 (0.1–3.0)	0.573	0.4 (0.06–2.4)	0.309	1.5 (0.3–7.0)	0.600	0.9 (0.2–5.1)	0.935
**Employment status**																
Employed	Ref	-	Ref	-	Ref	-	Ref	-	Ref	-	Ref	-	Ref	-	Ref	-
Unemployed	1.4 (0.7–2.8)	0.333	1.5 (0.7–3.5)	0.304	0.9 (0.4–1.8)	0.705	0.8 (0.4–1.9)	0.698	1.2 (0.6–2.4)	0.550	1.5 (0.7–3.5)	0.314	2.2 (1.1–4.3)	0.030*	2.5 (1.1–5.7)	0.023*
Other	1.3 (0.4–4.0)	0.618	0.8 (0.2–3.5)	0.798	1.6 (0.5–4.8)	0.402	2.0 (0.6–7.1)	0.289	1.3 (0.4–4.0)	0.616	1.4 (0.4–5.1)	0.620	1.2 (0.4–3.8)	0.699	1.3 (0.4–4.7)	0.649
**Distance travelled to the facility**																
5 km or less	Ref	-	Ref	-	Ref	-	Ref	-	Ref	-	Ref	-	Ref	-	Ref	-
6 km – 10 km	0.8 (0.3–2.2)	0692	1.0 (0.3–3.0)	0.959	0.7 (0.2–1.8)	0.420	0.8 (0.3–2.3)	0.639	0.8 (0.3–2.0)	0.565	1.1 (0.4–3.2)	0.916	0.9 (0.3–2.3)	0.771	0.9 (0.3–2.6)	0.828
11 km – 20 km	1.4 (0.6–3.6)	0.452	2.5 (0.8–7.6)	0.117	0.5 (0.2–1.3)	0.145	0.6 (0.2–1.8)	0.347	0.3 (0.1–0.8)	0.017*	0.5 (0.2–1.5)	0.200	0.7 (0.3–1.9)	0.513	0.8 (0.3–2.3)	0.662
Above 20 km	0.8 (0.3–2.2)	0.660	1.2 (0.4–3.8)	0.790	1.0 (0.4–3.0)	0.957	1.4 (0.4–4.7)	0.613	0.6 (0.2–1.7)	0.344	0.9 (0.3–2.7)	0.787	0.6 (0.2–1.5)	0.262	0.6 (0.2–1.9)	0.363

Note: All logistic regression analyses were performed at 95% CI.

UOR, unadjusted odds ratios; AOR, adjusted odds ratios; *p*, probability value; CMJAH, Charlotte Maxeke Johannesburg Academic Hospital; HCHC, Hillbrow Community Health Centre; SRH, South Rand Hospital; PHC, primary healthcare clinic; Ref, reference; km, kilometre; CI, confidence interval.

* , *p* < 0.05.

### Treatment-taking behaviour

Table 3 presents the reported treatment behaviour of the participants for the duration of receiving ART. There were more reports of treatment interruption whilst participants were on first-line treatment (*n* = 52). With respect to the reported treatment interruption whilst on second-line regimen, there was no distinct difference between the failing (*n* = 8) and suppressed (*n* = 5) groups. Both groups equally relied on themselves to remember to take their treatment (‘naturally [I] remember taking my pills [VLF, woman, 31 years]; [I am] experienced on remembering my time’ [VLS, man, 41 years]). Some of the reasons for interrupting treatment included stopping to take tuberculosis medication (‘yes, interruption due to TB recurrence’ [VLF, woman, 22 years]) and no medication availability whilst relocating (‘I once interrupted my treatment due to the shortage of drugs as I relocated in South Africa and I did not have a proper transfer letter’ [VLF, man, 38 years]). In the group with no reported second-line treatment interruption, more virologically suppressed participants (*n* = 21) listed using an alarm as a reminder. Several participants used the timeslots of popular local television programmes or the news (*n* = 14) as reminders to take their medication.

**TABLE 3:** History of antiretroviral treatment-taking behaviour.

Variable	First-line treatment			Second-line treatment					
	Treatment interruption	No treatment interruption	Did not report on treatment interruption	Treatment interruption		No treatment interruption		Did not report on treatment interruption	
				VLS	VLF	VLS	VLF	VLS	VLF
	52	91	6	5	8	67	56	6	7
**Medication alert**									
Alarm	21	53	2	1	2	21	13	2	1
TV	5	9	-	1	1	3	1	-	-
Memory	14	14	2	1	4	30	30	3	4
Family	7	9	1	1	-	2	4	-	-
Did not report	5	6	1	1	1	11	8	1	2
**Daily frequency**									
Once	16	37	1	-	2	5	1	1	1
Twice	33	52	4	5	6	60	55	5	5
Did not report	3	2	1	-	-	2	-	-	1
**Storage**									
Cupboard	31	57	3	4	7	39	35	3	5
Handbag	14	22	1	-	-	18	17	1	1
Cooler bag or fridge	1	1	1	-	-	-	1	1	-
Pill box	3	7	-	-	-	8	3	-	-
Other	1	1	-	-	1	-	-	1	-
Did not report	2	3	1	1	-	2	-	-	1

ART, antiretroviral treatment; VL, viral load; VLS, viral load suppression; VLF, viral load failure; TV, television.

Table 4 presents the bivariate and multivariate logistic regression analyses for ART-taking behaviour. There was no association between participants’ treatment-taking behaviour (i.e. medication alert, daily frequencies and pill storage) and VLF. However, participants who relied on their memory as a reminder to take medication (whilst on first-line regimen) were almost three times more likely to interrupt their first-line treatment than those who relied on an alarm (AOR: 2.6, 95% CI = 1.0–6.4, *p* = 0.042). There was no association found between medication alert or frequency of taking medication and second-line treatment interruption. Participants who used their handbag to store their medication were the least likely to experience second-line treatment interruption (OR: 0.2, 95% CI = 0.05–1.0, *p* = 0.054).

**TABLE 4:** Logistic regression analysis between virological failure, regimens (first-line and second-line regimen) and treatment-taking behaviour indicators in health facilities in Johannesburg inner city.

Variable	Virological failure				First-line interruption				Second-line interruption			
	UOR	*p*	AOR	*p*	UOR	*p*	AOR	*p*	UOR	*p*	AOR	*p*
**Medication alert**												
Alarm	1	-	1	-	1	-	1	-	1	-	1	-
TV	0.8 (0.1–4.6)	0.756	0.8 (0.1–5.5)	0.837	1.3 (0.4–4.4)	0.634	1.7 (0.5–5.7)	0.429	2.8 (0.4–19.1)	0.284	2.1 (0.2–18.2)	0.497
Memory	1.7 (0.8–3.7)	0.196	1.7 (0.8–3.8)	0.194	2.8 (1.2–6.6)	0.023*	2.6 (1.0–6.4)	0.042*	1.1 (0.4–3.3)	0.818	1.1 (0.3–3.7)	0.859
Family	1.9 (0.4–8.1)	0.398	2.1 (0.5–9.8)	0.339	2.7 (0.9–7.9)	0.069	2.7 (0.9–8.4)	0.080	0.7 (0.07–6.7)	0.764	0.5 (0.08–6.5)	0.592
Did not report	1.3 (0.4–3.6)	0.677	1.2 (0.4–3.6)	0.694	2.1 (0.6–6.8)	0.236	1.6 (0.5–5.8)	0.447	1.3 (0.3–5.0)	0.745	1.5 (0.3–6.9)	0.585
**Daily frequency**												
Once	1	-	1	-	1	-	1	-	1	-	1	-
Twice	1.4 (0.4–5.2)	0.604	1.6 (0.4–6.4)	0.479	1.7 (0.8–3.5)	0.153	1.7 (0.8–3.7)	0.162	0.3 (0.07–1.1)	0.060	0.3 (0.08–1.5)	0.149
Did not report	0.8 (0.05–11.3)	0.835	0.8 (0.05–13.3)	0.883	4.6 (0.8–27.9)	0.095	3.7 (0.6–26.1)	0.177	Empty	-	Empty	-
**Storage**												
Cupboard	1	-	1		1	-	1	-	1	-	1	-
Handbag	1.0 (0.5–2.2)	1.000	1.0 (0.4–2.2)	0.964	1.1 (0.5–2.5)	0.782	1.4 (0.6–3.1)	0.478	0.2 (0.05–1.0)	0.054	0.2 (0.05–1.0)	0.055
Cooler bag or fridge	0.5 (0.04–5.7)	0.577	0.5 (0.04–6.2)	0.592	3.5 (0.3–40.3)	0.312	3.8 (0.3–50.5)	0.309	1.9 (0.2–22.3)	0.602	1.9 (0.1–27.4)	0.606
Pill box	0.4 (0.09–1.5)	0.166	0.3 (0.08–1.4)	0.131	0.9 (0.2–3.7)	0.816	1.2 (0.3–5.5)	0.809	Empty	-	Empty	-
Other	Empty	-	Empty	-	3.5 (0.3–40.3)	0.312	1.7 (0.1–22.8)	0.678	Empty	-	Empty	-
Did not report	1.0 (0.2–5.2)	1.000	1.1 (0.2–6.3)	0.931	2.3 (0.5–11.1)	0.284	2.3 (0.4–12.2)	0.336	1.9 (0.3–11.3)	0.470	2.3 (0.3–16.9)	0.430

Note: All logistic regression analyses were performed at 95% CI.

UOR, unadjusted odds ratios; AOR, adjusted odds ratios; *p*, probability value; Ref, reference; CI, confidence interval.

* , *p* < 0.05.

### Recommendations from participants

The study participants made 175 recommendations for improving adherence (see Table 5). Coformulation in single tablets, only needing to take one dose of medication daily (preferably at night) and education about being adherent were listed as the most effective mechanisms to improve adherence on second-line treatment. Some examples of recommendations from participants include the following:

‘Education should be emphasised through adherence classes. Reinforce on the benefits of ART’. (VLS, female, 38 years old)

‘Availing a single-dose treatment for the second-line patients would enable them to adhere to treatment. Further ongoing education would also help’. (VLF, female, 39 years old)

**TABLE 5:** Participants’ perspectives on how adherence can be improved amongst second-line patients.

Recommendation	Number of people citing the recommendation
Coformulation in single tablets	55
One dose	46
Education	24
No recommendations	12
Injection	9
Psychosocial support	9
Smaller pills	7
No side effects	4
Clinic operating times	2
Counselling	1
Decreased frequency of treatment ingestion	1
Follow-up	1
Liquid	1
More research needed	1
SMS reminders	1
Trust patients	1

**TOTAL**	**175**

SMS, short message service.

Other recommendations included the development of injectable ART (*n* = 9, seven women and two men) and the provision of psychosocial support (particularly related to poverty and ensuring food supply):

‘Should consider addressing the psychosocial needs of patients on second line as they have to adhere to treatment but sometimes they do not have enough food to eat’. (VLF, female, 45 years old)

Single recommendations to improve adherence included treatment reminders (*n* = 1), additional counselling (*n* = 1) and minimising the number of times second-line regimen is taken per day (*n* = 1). One patient felt that healthcare workers trusting that their patients took their medication as prescribed would promote treatment adherence. Lastly, 12 participants did not have any recommendations (mainly because of not experiencing any pill-taking challenges), as shown in the following statement:

‘The current regimen is fine with me, therefore, I will suggest no change’. (VLS, male, 41 years old)

## Discussion

This study sought to describe and understand treatment adherence and possible treatment support interventions from patients receiving second-line ART.

It has been reported that relationship dynamics influence ART adherence and VLS in that being married or having a committed and supportive partner tended to foster an environment for better clinical outcomes in HIV-positive people.^17,18^ Studies from South Africa and the United Kingdom found that HIV-positive married individuals had better clinical outcomes compared to any other relationship status.^19,20^ Similarly, our study found that single and unmarried people living with their partners were more likely to be virally unsuppressed.

Not statistically significant but important for consideration in adherence strengthening, our study showed that being younger was a predictor of VLF, which was congruent with previous studies.^21,22,23^ We noted VLS in those participants who resided further away from the health facilities. This is not in agreement with findings of studies conducted in Uganda, Ghana and Burkina Faso,^24,25,26^ which reported that individuals who resided closer to a health facility were more likely to seek healthcare.

Late disclosure may hinder adherence or treatment support and subsequently yield poor clinical outcomes.^27^ Whilst the majority of participants (63%) disclosed their HIV status 1 week after diagnoses, about 28% took longer than 4 weeks to disclose. Early disclosure, particularly to a family member or partner, has been strongly associated with improved adherence.^8,28,29^ Disclosure to a family member or partner has been linked with adequate psychosocial support which in turn facilitates adherence to treatment.^8,29,30,31,32^ However, the findings of our study suggest that disclosure and dependence on a treatment supporter are likely not to produce desired adherence levels (and did not feature in the list of participant recommendations), indicating that disclosure and treatment support should be assessed in combination with other adherence strategies instead of as a single consideration or mechanism.^33^

Unsurprisingly, the more toxic the second-line multi-pill, and regimens requiring medication to be taken multiple times a day, were seen as significantly harder to take than a single tablet daily well-tolerated first-line regimen. These views were consistent with reports from other studies that attributed similar challenges to taking second-line regimen.^34,35,36^

Participants who did not interrupt ART mainly reported using an alarm as a reminder for taking their medication. This finding suggests the need to explore external reminder mechanisms for improving adherence in this setting, considering that about 15% of VLF participants reported not using any external reminders. Various studies have also found a trend towards better adherence amongst patients who used external reminders.^37,38,39^ In addition, our study showed that participants who used their handbags to store their medication were more likely to adhere to treatment. This finding is in line with other studies that have reported having a handbag to have pills all the time as the preferred ART storage by patients.^40,41,42^

Side effects are an important predictor of poor adherence, and cumulative toxicity associated with ART, especially in second-line regimens.^43,44,45^ We found that participants with VLF were more likely to have treatment-related side effects. Furthermore, those participants with side effects were more likely to be unemployed. Although this was not explored further in our study, various studies have reported that employed patients can manage their health and side effects better than their unemployed counterparts.^46,47,48^

Participants had ideas regarding drug formulation that may improve adherence. These included a fixed-dose combination, a dosage taken once a day and reducing the pill size. Furthermore, the participants suggested that education on the benefits of taking ART could improve adherence, whilst a few participants also suggested the implementation of injectable ART. Various studies have recommended similar strategies,^49,50,51^ with the effectiveness of some of these strategies being previously reported for first-line regimens.^51,52^

### Study limitations and strengths

This study had several limitations. Firstly, the study relied on participants’ self-reports, prompting a likelihood that socially desirable answers may have been provided. However, to control for this, information such as viral load, side effects and comorbidity was verified by checking participants’ medical records as part of data quality checks for the study. Secondly, the clinical measure for adherence considered viral load only. Finally, the sample might be small for the results to be generalised to all patients receiving second-line ART. However, the direction and size of effect were generally consistent, suggesting that the study findings may be robust despite these limitations.

## Conclusion

Participants on a second-line antiretroviral regimen had firm recommendations regarding improving adherence, largely focused on administration, reduced dosing and pill burden. The study results suggest the importance of improving patients’ knowledge about treatment and adherence and motivation to continue ART use despite the persistence of side effects. Drug manufacturers and health programmers must consider such recommendations as they modify and implement new ART regimens and programmes. Lastly, treatment support interventions recommended in this study need to be tested in practice to determine their efficacy for large-scale implementation.
